# Supplementary material for: Randomized experimental population-based study to evaluate the acceptance and completion of and preferences for cervical cancer screening
Source: PLoS One. 2024 Aug 9;19(8):e0306130. doi: 10.1371/journal.pone.0306130 (PMC11315343; doi:10.1371/journal.pone.0306130)
Supplement: Attachment — Submitted filename: Response-to-Reviewers-PLoSOne-PONE-D-21-23169.docx [file pone.0306130.s002.docx]

September15^th^, 2023.

**Excellent Sir**

**Farzad Taghizadeh-Hesary**

**Academic Editor**

**PLoS One**

**Dear Sir,**

We hereby forward the corrected and revised manuscript entitled "**Randomized experimental population-based study to evaluate the acceptance and completion of and preferences for cervical cancer screening**", authored by Marcela Vieira Lordelo, Cleyton Zanardo Oliveira, Luisa Aguirre Buexm, Rui Manuel Vieira Reis, Adhemar Longatto-Filho, Júlio César Possati-Resende, Fabiana Lima Vazquez, José Humberto Tavares Guerreiro Fregnani, for your consideration.

First of all, we would like to thank the reviewers of this study who provided valuable suggestions and very pertinent comments, thus contributing to improving the quality of our article. After a detailed analysis of the comments and questions, as well as the errors pointed out and suggestions contained in the opinions sent to us, the article has undergone some changes, which are indicated below.

Thank you in advance for your attention,

**Fabiana de Lima Vazquez**

*Researcher, Molecular OncologyResearch Centre, Barretos Cancer Hospital, Brazil*

RESPONSE TO THE EDITOR'S AND REVIEWERS' COMMENTS:

1. Please follow PLoS One's guidelines for the manuscript preparation (including the references).

The manuscript has been amended according to PLoS One guidelines including the references.

2. Please cite (at least) five relevant articles from PLoS One.

We have included relevant PLoS One articles in the area of the manuscript throughout the introduction and discussion.

- Lines 44-46 “The acceptance of Pap samples is extremely important to achieve positive results. Some limitations to performing a Pap sample have been previously reported, such as discomfort, shame, fear of pain, lack of time and lack of a reliable professional to perform the exam [4,5].” Referring to: PMID: 32614875

- Lines48-53 "It can complement other current methods, such as self-collection of vaginal samples, and can increase coverage in places that do not have easy access to Pap samples, since women in these areas will be screened only a few times in their lives and the high sensitivity of HPV testing is important and may increase the acceptability and effectiveness of screening programmes[6, 8-11]."Referring to: PMID: 32870941; PMID: 31042762;

- Lines 205-206 "Cervical cancer remains a global health issue, especially in less developed countries, where preventive exams have low coverage[21-23]." Referring to PMID: 29944694; PMID: 23049708.

3. The manuscript requires linguistic copy editing (please also submit the English copy-editing certificate).

The manuscript has been properly copy-edited and the language editing certificate attached in the PLoS One system.

4. Please revise the manuscript per the following reviewers' comments.

Below we respond to the reviewers' comments.

Reviewer #1 suggested the reformulations highlighted below:

1) Manuscript should be further revised in order to correct some typos and improve style.
Adequate. The text was revised and grammatically corrected, as well as structural adjustments.

2) To date, several lines of evidence support the possibility to use specific biomarkers to identify early stage cervical cancer and, in this way, offer a better prognosis to the patients. 3) This point deserves to be discussed, referring to: PMID: 28918603; PMID: 28466456.
Appropriate. Information included in the discussion of the manuscript.

- Lines 258-259 “It is worth noting that evidence currently supports the possibility of using specific biomarkers to identify cervical cancer early, providing a better prognosis for women [38-39].”

3) I would recommend to stress novel pieces of evidence about high-risk HPV-negative high-grade cervical dysplasia, which seems to have more favorable outcomes than patients with documented high-risk-HPV infection (PMID: 33514481) as well the potential role of pre-operative conization before radical hysterectomy for early-stage cervical cancer (PMID: 32320800).

Adequatetext. The topic has now been covered in the discussion.

- Lines 259-261 “Emerging data suggests that high-grade cervical dysplasia in HPV-negative patients has better outcomes than in patients with documented high-risk HPV infection [40].”

Reviewer #2 suggested the reformulations highlighted below:

1) There are a few typographical and technical errors that have been highlighted in the attached documents.

Appropriate. All the considerations pointed out by the reviewer have been corrected in the text.

2) Please do well to create sub-headings for the conclusion and others as stated in the attached document.

Adequate. Sub-headings created. The methodology includes the following sub-headings: Study Design and Sample Size, Inclusion and Exclusion Criteria, Data and Sample Collection, Ethical Consideration; and a Conclusion topic was also described.

Reviewer #3suggested the reformulations highlighted below:

1) Abstract-line 4: please add the "completion" to the study objectives.

Adequate. The information has been included.

- Lines 20-21: “The objective of the study was to evaluate the acceptance, preferences and completion of four screening methods.”

2) Line 80. Was "simple-random sampling" computer-based?

Adequate. The information was included.

Lines 66-67: “This was an experimental open-label study with a community trial conducted in the city of Barretos, state of São Paulo, Brazil”

3) Please improve the resolution of figures.

Adequate. The quality of the images was adjusted and sent via the PLoS One system.

4) 201-202: "The highest acceptance rate was found in the group assigned to undergo Pap sample collected at the hospital ambulatory, followed by the urine self-collection group and the group that allowed the participant to choose her preferred method". But all three groups had same acceptance rate of 100%. Please correct the sentence.

The acceptance rate is as described in the text, but the completion rate was not 100%, as we can see in the text of the manuscript and as shown in table 2.

- Lines 208-213“The highest acceptance rate was found in the group assigned to undergo Pap sample collected at the hospital ambulatory, followed by the urine self-collection group and the group that allowed the participant to choose her preferred method. The group assigned to have a Pap sample at the hospital showed the lowest completion rate; among the women who chose to go undergo this method, only 54% followed through to completion. Shame was the main barrier that women reported for not undergoing a routine Pap sample.”

- Line 214-217 “In the first group, all women who were asked to undergo a Pap sample at the hospital agreed to the screening by scheduling it for a day of their choosing. Therefore, a 100% acceptance rate was achieved. Regarding the completion rate, however, only 53.3% (16/30) went to the hospital to complete the test.”

5) The manuscript requires English copy editing.

The manuscript has been properly copy-edited and the language editing certificate attached in the PLoS One system.

6) In methods section, please mention that it was an open-label study.

Appropriate to the text.

- Lines 80-82: “In each stratum, the blocks (several houses) were numbered and randomly assigned to intervention groups by simple random computer-based sampling to ensure the appropriate number of women in each intervention group.”
